# Acknowledgment to Reviewers of *Children* in 2021

**DOI:** 10.3390/children9020195

**Published:** 2022-02-03

**Authors:** Children Editorial Office

**Affiliations:** MDPI AG, St. Alban-Anlage 66, 4052 Basel, Switzerland

Rigorous peer reviews are the basis of high-quality academic publishing, and thanks to the great efforts of our reviewers, *Children* was able to maintain its standards for the high quality of its published papers. Thanks to the contributions of our reviewers in 2021, the median time to the first decision was 20 days and the median time to publication was 40 days. The editors would like to extend their gratitude and recognition to the following reviewers for their precious time and dedication, regardless of whether the papers they reviewed were finally published:
A. Moreno GaldoKazuya KawamuraAamir JalilKeane WheelerAamir JeewaKeiko UchidaAaron HamvasKeisuke NagasakiAbdul Mohin SajibKeita TeruiAbdullah TolaymatKeith Da SilvaAbhishek Kumar SinghKeith PecorAdam CollisonKelly A. Rouster-StevensAdam Peter WagnerKelly CosgroveAdel A. MohamedKelly McGormAdel H. MansurKenji BabaAdeyinka AdebayoKerr GrahamAdhikarimayum Lakhikumar SharmaKerry-Ann GrantAdina BrahaKerstin GerholdAdina NegreaKerstin HeinAdolfo Laureano Bau-tista-CasasnovasKevin CahillAdrián CurtoKevin McCarthyAdrian Emil LazarescuKevin WeingartenAdrian LabarKeyla Vargas-RománAdriana CeciKhaled TrabelsiAdriana GrigorașKhaula KhatlaniAdriana MunteanKim GibsonAdriano CarottiKim Mooney-DoyleAdrien FrommerKimber SimmonsAdrienne Vanessa LevayKimberley HillAgathe MarcastelKimberly A. ChapmanAggarwal AnjaliKimberly A. Pyke-GrimmAgnieszka Kozioł-KozakowskaKimberly B. CastleAgnieszka PiekaraKin M. YuenAgnieszka Sobierajska-RekKippeum LeeAgnieszka SzymańskaKira PattersonAgnieszka ZachurzokKiran K. UpadhyayAgustín Llopis-GonzálezKiran NandalikeAhmed NasrKiran UpadhyayAida BairamKishore ChallagundlaAileen KirbyKitsaras GeorgeAimee PillerKlaudia SmolągAjay Pratap SinghKlaus LibertusAkihide OhkuchiKlaus RoseAkira TokiKnsuk ChauhanAlaa Hazem GaafarKnut Asbjørn Alexander ØymarAlan G. WoodruffKoen Van HoeckAlan RussellKoichiro TakagiAlan T. L. ChengKoji OtaniAlana L. BeresKong ChenAlba Martínez-GarcíaKonrad S. JankowskiAlbert TuKonrad ZuzdaAlberto CaprioglioKonstantin KrasagakisAlberto ChighineKonstantinos KotsisAlberto Gómez-MármolKonstantinos SidiropoulosAlberto González-GarcíaKoos Jaap Van ZwietenAlejandro Llanos-CheaKorey BoydAlejandro Mar-tínez-RodríguezKostas MichalopoulosAlejandro SerrabloKrishna Kishore UmapathiAlenka PavlicKrista SchroederAlessandra CostanzaKristen KrollAlessandra LuccheseKristin RispoliAlessandro CrocoliKristina DissingAlessandro FailoKristina GorsetaAlessandro MoroKristina RiosAlessandro NotaKristine Elizabeth Wood-wardAlessandro ParenteKristine M. KrajnakAlessandro PorrovecchioKrisztina MarthaAlessandro VittoriKriti PuriAlessio BellatoKrystyna KowalczukAlessio ContiKrystyna PawlasAlexander EngelKrzysztof JurekAlexander M. KepplerKumi Nagamoto-CombsAlexander NgwubeKun TangAlexander ParodyKun-Der LinAlexandra MafteiKurt HahlwegAlexandra RussellKurt WidhalmAlexandros PatrianakosKyle J. MorganAlexandru UliciKyle J. Van ArendonkAlfonso Castillo-RodríguezKyung‐Nam KohAlfonso PapparellaKyung-Sook BangAlfredo G. CasanovaL. Reid NicholsAli BoolaniLaila HugrassAlice GiontellaLampros FotisAlice GoisisLana B. KarasikAlice IndiniLara ReichertAlice NoblinLarissa TRUEAlice OrdeanLaura A. HanyokAlicia G. KachmarLaura Baena-GarcíaAlina SchartnerLaura C. DappAlina Simona RusuLaura C. Sánchez-SánchezAlison AndersonLaura DuncanAlison InnerdLaura E Miller-GraffAlison LaingLaura Fusar-PoliAlla KushnirLaura GilbertsonAllan GlanzmanLaura HouldcroftAllison DiBianca FasoliLaura KatusAllison SmithLaura RosAlmudena Alonso-OjembarrenaLaura StefaniAlok K. VermaLauren C. HyerAlthea Z. ValentineLauren E. HarrisonÁlvaro Astasio-PicadoLauren Joy LiebermanAlysse J. KowalskiLauren M. JanssonAlyx TaylorLauren StraussAmanat AliLaurent SuppanAmanda DeacyLaurie FishmanAmanda M. EvansLaurie L. MeschkeAmanda R. Hiles HowardLaurie SvobodaAmanda StoneLawrence Chukwudi NwabudikeAmelia DíazLeandra WoolnoughAmelia J. HessheimerLeandro TeixeiraAmina MunirLeeann PavlekAmir Khorram-ManeshLei WanAmit ChandelLeila HojatAmna QasimLeon Garland ColemanAmy ConradLeonard G. FeldAmy JohnsonLeonardo CesanelliAmy L DrendelLeonardo Emberti Gial-loretiAmy SmithLesław SzydłowskiAn JacobsLeticia E. SewaybrickerAna Álvarez MuelasLeyre MadariagaAna CoelhoLi HeAna IvaniševićLi-Ang LeeAna KatusicLiang YeAna Luísa Moreira CostaLiang-Jen WangAna Maria Vazqquez Cas-aresLibor VelíšekAna Martínez-PampliegaLicínio MancoAna ReulaLidia GavicAna Rita MatiasLidia PerencAna SaniLigia LimaAnabela Cruz-SantosLihong HuangAnahi Alba De La FuenteLihong JiaAnandharajan Rathinasa-bapathyLiliana Aguayo-MarkesAnant VatsayanLiliana Szyszka-SommerfeldAnastasia Kerr-GermanLiliane SaidAnca MesarosLille KurvitsAnca UngurianuLina BegdacheAndre WirriesLina MichalaAndrea AbateLinda FranckAndrea BehrmanLinda J. Larson-PriorAndrea BorghesiLinda MilnesAndrea ButeraLinda SangalliAndrea ColemanLinda SaraivaAndrea CostantinoLinda SizibaAndrea CuvielloLinda VisserAndrea Del GiudiceLingxiao XieAndrea Di CataldoLingyun JiAndrea KattahLin-Ya HsuAndrea LauferLira YuAndrea MartinuzziLisa C. Bowman-BowenAndrea Martín-VacasLisa T. JansenAndrea ScaramuzzaLise HestbækAndrea SchmeddingLiudmila LiutskoAndrea TendasLivia GaravelliAndrea TrombettaLivia OttolenghiAndreas JaegerLiz McDowellAndreas KühnapfelLoc DoAndreas MeinzerLorås LennartAndreas TrotterLoredana AmorosoAndrés García-GómezLorenzo FaggioniAndrew C. PickettLorenzo MariniAndrew ClarkLorenzo ZaffiriAndrew GrayLori A. DevlinAndrew J. MurphyLori Elmore-StatonAndrew JinLori WienerAndrew K. GormleyLorraine Kelley-QuonAndrew KaczynskiLouis BezuidenhoutAndrew M. DylagLourdes Martinez-NietoAndrew PitchfordLourdes PrietoAndrew SchmidtLuca BedettiAndrew ShinLuca BonadiesAndrew ThompsonLuca De SimoneAndrzej BrudnickiLuca DenaroAneeza W. HamizanLuca EspositoAnette Ramm-PettersenLuca MagistrelliAngela ConejeroLuca PierantoniAngela CostabileLuca Redaelli de ZinisAngela GallerLucas BrandaoAngela JacquesLucas TrepsAngela M KolenLucia MiligiAngela MastronuzziLucia MolettaAngela TitmussLucia SchiavonAngeliki AngelidiLuciana Paula SamoranoAngelo AulisaLuciane R CostaAngelo BarbatoLucie FinezAngelo V. VasiliadisLucie JurekAnh T. P. NgoLucie MacchiAnirudh SharmaLudovica PascaAnja IvicaLudovico DocimoAnja KühnelLuigi BruscianoAnja SteinbachLuigi Dall’OglioAnjali A. DixitLuigi De GennaroAnn MastenLuigi MazzoneAnna AxelinLuigi MeccarielloAnna B. FuksLuigi SantacroceAnna BednarekLuigi TarantiniAnna BiernasiukLuigi TitomanlioAnna BogdanovaLuís DelgadoAnna CamporesiLuis Manuel Mota De Sou-saAnna CzarneckaLuis Mariano EstebanAnna Di SessaLuis Peña QuintanaAnna Gagat-MatułaLuísa Bandeira LopesAnna I. R. Van Der MiesenLuisa Losada Losada PuenteAnna Kaisa NiemiLukas D. LandeggerAnna Katharina HollLukas F. HeilmannAnna Kathrin HellLukas P. MilederAnna LewandowskaŁukasz M. MilanowskiAnna ManelisŁukasz TomczykAnna Maria CostaLuke J. NeyAnna Maria HibbsLuminita PaduraruAnna NilssonLydia E. DevenneyAnna PilutinLydia Gabriela SpeyerAnna PriceLyndsey MuehlingAnna RozensztrauchLynn Clark CallisterAnna Sophie RommelMacarena Lozano-LorcaAnna StarzyńskaMaciej GawęckiAnna SurdackaMaciej JedlinskiAnna Valeria SamarelliMack ShelleyAnna WłochMaddalena Fabbri-DestroAnnalisa PaceMadelyn LabellaAnne J. SpaansMaegan CalvertAnne ParkinsonMagdalena BaymakovaAnneloes van BaarMagdalena Sycinska-DziarnowskaAnnette BronsMagnus SvartengrenAnnika AnderssonMa??gorzata OsmolaAnnio PosarMajid Mufaqam Syed-AbdulAnn-Marie Malby SchoosMaki UrushiharaAnouschka FoltzMakoto NagoshiAnqi ShenMalgorzata GrzesiakAnthony C. FaberMalgorzata KosteckaAnthony HerbertMałgorzata StachońAntoine GiraudMalgorzata WasniewskaAntonella FrassanitoMalgorzata Witkow-ska-ZimnyAntonella GrittiMałgorzata ŻukAntonino MorabitoMamoru AyusawaAntonio CorselloMana RaoAntónio FerrazManish JainAntonio González-BulnesManlio SantilliAntonio Jesús Mar-tínez-OrtegaManoja Kumar DasAntonio NarzisiManolis AdamakisAntónio NogueiraMANUEL Coheña-JimenezAntónio QueirósManuel Joaquín De No-va-GarcíaAntonio ScaranoManuel KroneAntonio-Javier ChamorroManuel Pabón-CarrascoAntonio-Jesús Marín-PazManuel SorianoAntonios K. GounarisManuela AlbisettiAnu ChackoManuela SimonatoAnup S. PathaniaManuela VerissimoAras BozkurtMara RiminucciAravind ThavamaniMarc LochbaumArimatias RaitioMarcela Dávila LópezArine VliegerMarcela Martín-Del-CampoAriuntuul GaridkhuuMarcelo WajchenbergArkadiusz ZurawskiMarcin CzopArkaitz CastañedaMarcin MorońARMANDO D'AngeloMarcin StraburzynskiArmin BadreMarco AtteritanoArmin FinkenstedtMarco CarliArnard Van Der ZwanMarco CarotenutoArne LowdenMarco CattaliniArne Silfverdal SvenMarco CroccoAroa Tardáguila-GarcíaMarco GötzeArpit AminMarco LottiArpitha ChiruvoluMarco MeucciArshi KhanamMarco Ramirez-OrtizArshid M. MirMarco Sapienza SapienzaArul ChandranMarco Stefano DemarchiArun SwaminathanMarcos Mecías-CalvoAsgeir MamenMarek ManderaAshlesha KaushikMarek UssowiczAshraf S. HarahshehMargaret B. MitchellAsif H. KhanMargaret F. KeilAskin GulsenMargaret ThomasAsterios KampourasMargarida Gaspar de Ma-tosAtbin DoroodchiMargarida VieiraAthanasia PrintzaMargarita Gozalo DelgadoAthanasios KaditisMargarita KaushanskayaAtif Ali AhmedMargherita ProsperiAudrey AdjiMaria Blanca Salinas-RocaAugust WrotekMaria Cecilia Mendon-ca-TorresAugustine W. KangMariá CernadaAurora ParodiMaria ContaldoAurore GuyonMaria Da Gloria Teixeira De SousaAussems SuzanneMaria Elena LunatiAviv ShmueliMaria Elisabetta Baldas-sarreAyataka FujimotoMaría Fernán-dez-HawrylakAyla YagdiranMaria GacekBabatunde RajiMaria GarraffaBadicu GeorgianMaria GrauBahaaldin AlsoufiMaria Grazia CagettiBallambhattu Vishnu BhatMaría José García-PolaBansod Yogesh DeepakMaría José Soto MéndezBarbara BaranowskaMaria José SousaBarbara ChoromańskaMaria LucaBarbara JuenMaria ManzanaresBarbera J. BoucherMaria MarinoBarry WrightMaria Mendoza-MuñozBart RuttemanMaria Michelle Papami-chaelBart VisserMaria Oliva-HemkerBarthelemy ToselloMaria Paola BonasoniBartosz Jan MusielakMaria Paula SantosBeata Bujak-GizyckaMaría Pilar Pecci-LloretBeáta HubkováMaria Pokorska-ŚpiewakBeata Szulc-MusiołMaria Salem IbrahimBeatriz Beltran-NavarroMaria SerrattoBee K TanMaria ShindovaBehnam KeshavarzMaria Teresa TrentinagliaBenedetta RagniMaría Victoria De GálvezBenedetto VitielloMaria-José EzeizabarrenaBeńed́icte ManouryMarian Baker-mans-kranenburgBenjamin J. LandisMarian LevyBerger NinaMarián Pérez-MarínBergsma AtzeMarian Sorin PaveliuBernhard HaidMariana Di LorenzoBernie CarterMariana FloriaBertil RydenhagMariana-Daniela Gonzá-lez-ZamarBeth K. ThielenMarianna BonnertBettina HieronimusMariantonietta FrancavillaBeverly KingstonMarie BičíkováBhola PradhanMarie BlackmoreBhola Shankar PradhanMarie FriedelBianca LinkMarietta KissBianca Weinstock-GuttmanMariia MatveevaBibhuti Bhusan DasMarijke GordijnBibhuti DasMarika GuggisbergBilal Alashkar AlhamweMarina LaborBin LiMário EspadaBin-Bin ChenMario GiordanoBinod KumarMario Mendoza-SagaonBirgit B. Ertl-WagnerMarion MateosBirgitta JälevikMarios SpanakisBjoern VogtMariusz DuplagaBlake JonesMark A. ConnellyBlanca ScheijenMark A. GottliebBo ChawesMark D. SimmsBo JinMark DzietkoBO OlusanyaMark EttenbergerBob PhillipsMark GottliebBonney ReedMark HaddadBoris ZernikowMark MiddletonBoyoung HongMark R. ProctorBrad FarrantMark ReybrouckBradley KellerMark ScherBrandon NathanMarko KavčićBrenda GolianuMarko ZdravkovićBrenda P. WigginsMarkus JuonalaBrian J. DlouhyMarleen M. RenardBrian LittlechildMarta StarnoniBrian ScottolineMarta TremoladaBrian ShawMartha F. MherekumombeBrian SwannMartie GillenBrianna YamasakiMartijn J.J. FinkenBritt GustafssonMartin L. MetzelderBrittany J. JohnsonMartin MüllerBruce DickMartin SalöBruno ChrcanovicMartin WeltmanBruno José Nievas SorianoMartina MicaiBryan KolbMartina OzbičBushra AfzalMarvin MillerC. Meghan McMurtryMary Ellen McCannCaitlin Martin-WagarMary K. LynchCalum BennachieMary KhetaniCameron K. TebbiMary Lauren M. NeelCameron PeersMaryna Krawczuk-RybakCamila B. WaltersMarzia LazzeriniCamila WaltersMasaaki MoriCamille BrehinMasafumi NiiCamillo RicordiMasayuki OzakiCao Ying-tingMassimiliano FilostoCarin Andrén AronssonMassimo A. PadalinoCarina VenterMassimo ConteCarl J. BassiMassimo MapelliCarl J. LavieMateusz JankowskiCarl LoMathieu BergeronCarl StafstromMathieu NacherCarlo BizMatt OwensCarlo BuonannoMatteo ChiappediCarlo DaniMatteo Di NardoCarlo FuscoMatteo RitrovatoCarlo VascottoMatthew D. DunCarlo ZancanaroMatthew D. EliasCarlos CoboMatthew MoormanCarlos LaranjeiraMatthieu GuemannCarlos Martin LlorenteMattia ManfrediniCarlos SisniegaMattias SchaeferCarlos ValienteMaura KepperCarlton DampierMaura MassiminoCarmen CasanovasMaureen Daly GogginCarmen F. Navarro-PérezMaureen E. LyonCarmen Pérez-RodrigoMaurizio EliaCarmine NovielloMaurizio GenteCaroline LewMavis DuncansonCaroline ParkerMaximo VentoCassandra S. DiepMay BisharatCaterina DaporMay YehCatherine J. SinnottMaya Leventer-RobertsCatherine L. ZemanMaylis BraunCelestyna GrzywniakMayri Sagady LeslieCesar A. SoutulloMazhar ZoubiCésar Calvo LoboMd Nabiul IslamChad A. GlennMegan BlackburnChad CriggerMeghan S. ZimmermanChang Won ChoiMeghashyam BhatChani TraubeMelissa A.E. LawsonChaoling ChenMelissa LuigCharles C. LeeMelissa N. AndersenCharles ObergMelissa Spezia FaulknerCharles TaiebMelissa S.W. YamauchiCharles ZeanahMeng-Che TsaiCharli ErikssonMeng-Yu WuChasity BrimeyerMercedes FernandezChaya S. MoskowitzMeropi ToumbaChelsea PelletierMette RaunkiaerChenghui LiM.F. AhamedChethan SampathMhd Louai ManiniChia-Che LeeMichael AbsoudChiara PanicucciMichael Ben-AvieChiara PeciniMichael CottonChiara SacchiMichael FreemarkChiarella GiuseppeMichaël HoferChiarella SforzaMichael J. McNeilChien-Huei HsuMichael J. RutterChien-Hung LeeMichael MedingerChiharu OtaMichael MeyerChing-Jin ChangMichael MoritzChi-Nien ChenMichael NarveyChintan GandhiMichael P MeyerChisa ShinsugiMichael SchwaigerChloe BedardMichael SmithChris RossiterMichael StarkChrista PereiraMichael StephensChristiaan Van BergenMichael WatsonChristian ApitzMichael WeissChristian BeltzerMichał BronikowskiChristian HirschMichał BrzezińskiChristina BahrsMichal CzaplaChristina GallinatMichał LatalskiChristina KriegelMichal SarulChristina LamMichel HabibChristine MottMichele CalleaChristoph BührerMichele D'AttilioChristoph HärtelMichele Di CosolaChristophe ChardotMichele D.M. LombardoChristopher CollinsMichele FinottiChristopher FrancisMichele MadduxChristopher S. CooperMichele PolettiChristos K. Yiannakopou-losMichelle BatthishChrystal GaertnerMichelle de HaanChui Chan HonMichelle Thornton AdlerChunbin ZouMieszko WięckiewiczChung-Ho E. LauMiglė BacevičienėChung-Ying LinMiguel AlsinaCilla SöderhällMiguel Ángel PulidoCindy CrawfordMiguel Ángel Rojo Rojo TiradoCinzia MasperoMiguel R. Pecci-LloretCiro EspositoMiguel Ramón Pecci-LloretCiro VillaniMiguel RebeloClaire ForrestMihajlo B. JakovljevicClara Yieh Lin ChongMiho KawaidaClaude ScheuerMika L. NonoyamaClaudia CalvanoMikko HallmanClaudia MaehlerMikolaj DabrowskiClaudia Ollauri-IbáñezMilan UrikClaudia Quaiser-PohlMilojevich Helen MCláudia Regina Furquim De AndradeMin Sun KimClaudia Talero-GutiérrezMing Horng TsaiClaudio ImperatoriMing LimClifton AddisonMing Wai WanConsolato SergiMingbang WangConstantinos Alexandros DemopoulosMing-Chou ChiangCorey StiverMinh Hoang NguyenCori L. DainesMinna AnttilaCorina PienarMio EbatoCorinna MartarelliMiranda A.L. Van TilburgCornelius GroenewaldMiranda van TilburgCorrado LupoMiriam Romero-LópezCorsino Rey GalanMiriam Sánchez-SanSegundoCostanza TognonMiriam ZacchiaCrisanta-Alina MazilescuMirja HirvensaloCristian LieneckMirjana TurkaljCristian ZeniMiro JukićCristina Anna Maria Lo IaconoMiroslava PřidalováCristina CalvoMirosława Szark-EckardtCristina FloreaMirto FolettoCristina Ioana BicaMitchell EversCristina Sánchez-RomeroMitsuru SekiCristoforo PomaraMittal JasoliyaCynthia KarlsonMohammad Abdelmotaleb Al-MotlaqD. GrothuesMohammed DiykhDaan DierickxMojtaba KavianiDabor ResiereMomcilo JankovicDac MaiMónica AznarDai ChungMonica MoneaDainius K. PûrasMonika Dmitrzak-WęglarzDamiano FormentiMonika FryszDamiano MeninMonika HagaDamien C. WeberMonika Łopuszańska-DawidDan DorobantuMonsurat LawalDana BadauMontserrat IzquierdoDanguole Ceslava RugyteMoonBae AhnDaniel B BlattMorgan A. FinkelDaniel HolzingerMorie Kristen P.Daniel J. ScherzerMosarrat J. QureshiDaniel KlotzMostafa WalyDaniel MissailidisMuhammad ChutiyamiDaniel P. KohenMukherjee DipankarDaniel StoecklinMulazim Hussain AsimDaniela CodrichMuneaki MatsuoDaniela DibelloMyriam Bickle GrazDaniela Pia Rosaria ChieffoMyriam Van WinckelDaniela RagoNadine ChoueiterDaniela RenziNahed M. Abdel-HaqDaniela ŠincekNahla ZaghloulDaniela TestoniNahum Mendez-SanchezDaniele CavaleriNa-hyeon (Hannah) KoDaniele FarsettiNaiara OzamizDaniele PironiNaokado IkedaDaniele PiscitelliNaoko ItoDaniele TrevisanutoNaomi MatsumotoDanielle FriedmanNaomi SwellerDanielle GreenbergNariae Baik-SchneditzDanila De VitoNassib B. BuenoDanny JazmatiNatalia BudaDanuta Januszkie-wicz-LewandowskaNatalia MazanowskaDaphne MerkusNatalia Ukleja-SokołowskaDariusz Wojciech Mazur-kiewiczNatalia V. BeloborodovaDarko AntičevićNatalia ZdanowskaDave Van KannNataliya UshevaDavid BeckerNataša VardaDavid BellNatascia RinaldoDavid BerbrayerNathalie ClaessensDavid BeversdorfNathalie GodefroidDavid BoothNathan BlueDavid BraunNathan M. NovotnyDavid CatelaNathaniel Kendall-TaylorDavid ChampionNatoshia CunninghamDavid E. MandelbaumNatoshia R. CunninghamDavid G IngramNavin PintoDávid LíškaNayanjot Kaur RaiDavid MandelbaumNeelakanta KanikeDavid MartinNeeltje Van Den BedemDavid R. BassetNeha KumbhatDavid R. RepaskeNeil FinerDavid Ramiro-CortijoNelson AlphonsoDavid S. CooperNevena ManevskaDavid S HurstNicholas RileyDavid Sancho-CantúsNicholas ShamanDavid Sanz-RivasNicklas Heine StaunstrupDavide BernasconiNicola KleinDavide CavagnettoNicola ZampieriDavide LeardiniNicolae MironDavide LugliettoNicolas J. PejovicDavide PisaniNicolas ScellesDavide RibaldoneNicolas VignierDawid SzwedowskiNicole C. MalloryDe Jesus Rojas WilfredoNicole DempsterDébora Regina De Paula NunesNiels WedderkoppDeborah MoncrieffNigussie Simeneh EndalewDebra C. VigilNiki JurbergsDebra PeplerNiko KacirotiDeepak JainNikoleta PrintzaDeepika SankaranNina Jakhelln LaugenDelia HorhatNiranjan VijayakumarDemin CaiNishant Ganesh KumarDenis CozziNishel Mohan ShahDenise BotheNishitha PillaiDenise Paschoal SoaresNitya BakshiDenise SharonNivine I. HanachDevendra DusaneNoah MoruzziDhaval ChauhanNobuo KanazawaDiana RamNobutaka KuriharaDiana RichterNoor KazimDiane Levin-ZamirNorihiko KikuchiDiego A. BonillaNorihiro NishidaDimitrios PanagopoulosNorman JungeDimitrios PapadimitriouNozomu HanaokaDimitrios PapadopoulosNuno HermannDimitris ZacharoulisNuno LoureiroDing-Wen (Roger) ChenNuno MoraisDion Tik Shun LiOana Falup-PecurariuDirk BasslerOctavian PostolacheDirk DhosscheO'Halloran KatrinaDomenico LioOkada NaohiroDomenico Umberto De RoseOlaf GefellerDomingo Gonzá-lez-LamuñoOlga N. BerdinaDominika MazurkiewiczOliver HenkeDominika SkolmowskaOliver HirschDominika WilczyńskaOliver MannDominique VervoortOlivier DanhaiveDonald H. ShaffnerOlof SandgrenDonatella Di CorradoOmar HikmatDonato RiganteOrsola Di MartinoDong In SuhOsamu NomuraDong-Hyun KimOscar HerreroDora GosselinOvidiu Popa-VeleaDora Isabel Fialho PereiraOxana DobrovinskayaDoreen SchmidlP J RobinsonDoris RusicP. Syamasundar RaoDorota SikorskaPablo A. Cantero-GarlitoDorota WójcikPablo Hernández-AlonsoDorothy E. DowPaige Terrien ChurchDouglas L. HillPam BrydenDror PaleyPam JarvisDugald SeelyPam ThomasonDulce Romero-AyusoPamela Di GiovanniDuncan Daniel R.Panagiotis KorovessisDustin WallacePanagiotis MallisEberhard LurzPanigrahi ArunEboni LancePanoraia SiafakaEdel Noriega-ÁlvarezPantelis PerdikarisEdgar De AlmeidaPaola BucciEdmond BowersPaola DamianiEdoardo SpinaPaolo FabbriniEduardo J. SimoesPaolo ImmovilliEdward C. KirkpatrickPaolo MontaldoEdyta ŁuszczkiPaolo RebullaEero LauhkonenParis BinosEffrosyni ManaliParker TichkoEfraín Santiago-RodríguezParul DayalEfrosini PapaconstantinouPascale DucheEgbert HertingPasqua PiemonteseEgidio BarbiPasquale FarsettiEinar ArnbjörnssonPatricia A. RichardsonEirini KostopoulouPatricia Palomo LópezEiso HiyamaPatricia SheinerElaine BoylePatrick D. EversElaine F RemmersPatrick JayEleftheria SamaraPatrizia ZaramellaElena AmaricaiPatrycja Sosnow-ska-SienkiewiczElena Crehuá-GaudizaPatryk LipinskiElena LadasPaul HibbardElena MaestriniPaul J. CritserElena PredescuPaul MonagleElena Sinkiewicz-DarolPaul MortonElena TarcaPaul NathanElena-Daniela GrigorescuPaul Nathan GoldwaterEleonora RosiPaul R. CarneyElio BignardiPaula Fernández GarcíaElisa BarbieriPaula PeixeElisa Lopez-DoladoPaulina KrawiecElisa MancinelliPaulina MetelskaElisabet A. ForsumPaulo PalmaElisabet ForsumPavel KotlarskyElisabet Sánchez-RodríguezPawel Antoni KolodziejskiElisabeth AndriePayam Payam ValiElisete DiogoPayam ValiElizabeth A FialkowskiPaz SuarezElizabeth A. HerrupPedro DiasElizabeth A. O’ConnorPedro Hidalgo LopezosaElizabeth AsztalosPedro M Valero-MoraElizabeth HillPedro MeloElizabeth J. HalsteadPedro MunueraElizabeth ReifsniderPedro Valdivia-MoralElizabeth VillegasPeer Van Der HelmElizete Aparecida LomaziPei-Shan HoElke KniselPentti NieminenElodie CharbonnierPer Morten FredriksenElvin Thomaseo BurtonPere Lavega-BurguésElżbieta SitarzPermani C. WeerasekaraEmanuel RaschiPetca Razvan-CosminEmanuela Gualdi-RussoPeter Anto JohnsonEmanuele ChisariPeter BaderEmanuele CozzaniPeter BaggettaEmer M. BarrettPeter BottenbergEmilee FlynnPeter F. W. RosierEmilia OgodescuPeter GänglerEmily K. SchaefferPeter H. BaenzigerEmily K. SchworerPeter HauserEmily K. WiecekPeter JonesEmily Shaffer-HudkinsPeter KarachunskiEmma FisherPeter MatteiEnikő FöldesiPeter N. JohnsonEnrique UrreaMendozaPeter N. SchlegelEreny GobrialPeter PaalErez Bar-HaimPeter S. WalkerEri MatsubaraPeter SimmEric A. SribnickPeter W. ChoateEric L. ScottPeter ZimmermannEric M. ChinPetr TvrdikEric OhumaPetra Corianne VinkeErica L. T. Van Den AkkerPhilip A. PowellErich RutzPhilip BufflerErik BootPhilip J. YoungErika HernandezPhilip MayErin TurbittPhilip SarajlicErling LundevallerPhilipp DeindlErmelinda De MeoPierluigi MarzuilloErnest G. ChanPiero FarruggiaErnest TyburskiPiero PavoneErnestas ViršilasPierpaolo MincaroneErnesto LevaPietro FerraraEryk LatochPietro MontemezziEsther Monika SpeerPietro MuratoriEsther Moraleda SepúlvedaPietro SalvagoEstrella RodriguezPietro SmirniEugène A.A. RameckersPietro VajroEugene KimPiotr AdamczykEun Jae KoPiotr DuchnowskiEungkwon PaePiotr Kalici??skiEva Aguilar-MediavillaPiotr LeszczyńskiEva BergstraesserPiotr MorasiewiczEva ClaussonPiotr SzymaniecEvalotte MöreliusPocecco ElenaEvangelia AntoniouPo-Chang HsuEvangelos AxiotisPo-Chin YangEvangelos FradelosPragya Sharma GhimireEvi VerbecquePramod K SahuEwa CieslikPranoot TanpaiboonEwa DudzińskaPrasad NalabothuEwa GieysztorPriscila (Caçola) TamplainEwa HenckelPritesh JainEwa RzońcaPriya Iyer-EimerbrinkEwa SkarżyńskaPriya MorjariaEwa WronaPriyanka MishraEwell S. RoachProleta DattaFabia FrancoProwse KatherineFabienne LigierPui Fong KanFabio CampodonicoPui Man Rosalind LaiFan-Fen WangPuspa Raj PantFang LiuQi FangFani LadomenouQi JinFarya PhillipsQiang ChenF??bio Saraiva FlôresQiaoyi ChenFederica AltieriQinxin ShiFederica BevilacquaQoot AlkhubaiziFederica CorradiR. Rodney HowellFederica Di SpiritoRachael TaylorFederica PorcaroRachana ShahFederico FerrariRachel D. VanderlaanFederico Martinón-TorresRachel K. PetersonFelicitas BidlackRachel M. BurkeFelix Javier Jimé-nez-JiménezRachel Neff GreenleyFélix Marcos TejedorRachel RundleFelix NeunhoefferRadosław KempińskiFernando Barragán MederoRadovan BilekFernando C. BaltanásRaegan MurphyFernando GarcíaRafael DenadaiFernando Gonzalez-AlonsoRafal NowakFerruccio TorselloRaffaele OrnelloFilippo MiglioriniRaffaele ScuratiFirst Name Last NameRaffaele SerraFlorence CarrouelRaffaele VitielloFong RoselineRaffaella BrugnoniFotini VenetsanouRagnar G. BjarnasonFouad S. SalamaRajamma MathewFrances CooleyRajdeep PooniFrancesca BorzìRalf HusainFrancesca De BernardiRalf WeigelFrancesca GarofoliRama Sashank MadhurapantulaFrancesca GoriniRamu VadukapuramFrancesca LatinoRandall T. LoderFrancesca SantamariaRanjit PhilipFrancesca ZaraRanjith KamityFrancesco BaccelliRaquel M. GuevaraFrancesco BellomoRaquib HasanFrancesco BiancoRasmus GustafssonFrancesco CadarioRaúl Jiménez BoraitaFrancesco DeleoRaúl Soto-CámaraFrancesco InchingoloRaul Zamora-RosFrancesco LeonRaymond OyenFrancesco LuparielloRebecca DistefanoFrancesco PisanuRebecca HamblinFrancesco TandoiRebecca LondonFrancesco ValituttiRegan FoustFrancis RiesRegina GairalFrancis TayieReinald BrunnerFrancisco Alcantud MarínReisinger Debra L.Francisco Al-cantud-Mar??nRemco J. MolenaarFrancisco Gonzalez-SalaRemo MombargFrancisco Pablo Holga-do-TelloRemster EricFrancisco Sánchez‐BuenoRenata GallagherFrancisco Teixeira Da SilvaRenata RutkauskaiteFrancisco ValeRenata ZadroFrancisco Villegas LirolaRenato GondarFranco GiraudoRenuka T. MenonFrank JochumReto HuberFrank M. SchiedelReza AbdollahipourFrank Thomas RiedeReza Akhavan-SigariFrederik J.A. DeconinckRhodri LloydFriederike BarthelsRhoikos FurtwänglerFulvio PlesciaRicardo Javier Marti-no-AlbaG. Allen FinleyRicardo Martino AlbaGábor GazdagRiccardo CappatoGabriel Angel Martos MorenoRiccardo GobbiGabriel SamascaRiccardo IzzoGabriele MascheriniRiccardo MasettiGabrielle Ten VeldeRichard H. Parrish IIGaëlle SauthierRichard LichensteinGaetano CairoRichard PauliGaetano ChiricoRick FerkelGail BrottmanRiitta A. NiinimäkiGang ChenRinze NeuteboomGang PengRishika SakariaGangaram AkangireRita AgarwalGarey NoritzRita CeruttiGarsiya Ekaterina Rob-ertovnaRita Gobet GobetGary M. WoodsRita Marie RyanGary MaRitesh ChimoriyaGary SteinmanRobert A. HirstGaskins DorotaRobert E. ShaddyGautam RameshRobert H. RiceGemma Moya-GaléRobert M. SiegelGeoffrey MadeiraRobert McMillenGeorg HoffmannRobert S. WareGeorge AntonogeorgosRobert StrickleyGeorge TarabulsyRobert W ArnoldGeorgios DimakopoulosRoberta BalestrinoGeorgios MitsiakosRoberta BattiniGerald FerrettiRoberta Di GiacomoGerard CortinaRoberta IaconaGerda MeijlerRoberta PalmourGerda van Wezel-MeijlerRoberto ChimenzGère Sunder-PlaßmannRoberto Lo GiudiceGerri MattsonRoberto MinieroGertjan Jan A. DriessenRoberto Ortiz-MovillaGiacomo BriscaRoberto Sanchez-CabreroGiacomo GaribottoRoberto W. Dal NegroGiacomo PapottoRocío De Diego CorderoGianluca EspositoRocio Martin - ValeroGianluca M. TartagliaRocio Palomo-CarriónGianluca TestaRoddy HiramGianni Di GiorgioRodrigo Elías ZambranoGilberto B. FischerRohan SharmaGina M. LockwoodRohit S. LoombaGina R. RosichRoland MooreGina ToméRolf SchlößerGiorgia VaralloRolf Van DickGiorgio D. KotzalidisRomina FucàGiorgio Lo Lo GiudiceRonald HoggGiovanna RiccipetitoniRonald R. SeeseGiovanni BoscarinoRonan MurphyGiovanni FilocamoRonit A. WollsteinGiovanni GalloneRonnie LevinGiovanni La CannaRosa Maria Mendiza-bal-EspinosaGiovanni MartinelliRosa Martha Meda LaraGiovanni ParenteRosanne A. CouttsGita WahiRosaria BarracanoGitanjli AroraRosario PastorGiulia BardiniRoss DrakeGiulia PurpuraRoxana Maria NemeșGiuliana BontàRoxana SiposGiulio CalcagniRoxana StegaroiuGiulio DistefanoRoy McConkeyGiuseppe CinalliRubén Navarro PatónGiuseppe G. LoscoccoRubén Sánchez-GómezGiuseppe LauritiRuchire Eranga WijesingheGiuseppe MaggioreRüdiger GreinertGiuseppe MartuccielloRudolf K. BraunGiuseppe ProcinoRudolf KorinthenbergGlenn C. BlomquistRui LiGloria Jiménez-MarínRui NevesGloria PelizzoRui PoinhosGobbo FrancescaRuiz-Herrera NoeliaGonzague JourdainRuri FameliaGos MonikaRu-Si ChenGrace Ellen BrannonRussel S KirbyGrace Mang Yuet MaRussell HoppGrazia BossiRussell J ButterfieldGregorio-Hernández Re-becaRussell K. WooGregoris SimosRuth S. HwuGregory FirthRyan BurnsGregory Neal BarnesRyan CallahanGreig TaylorRyan W. WalkerGrzegorz BazylakRyszard GrendaGrzegorz PituchaS. BeaudoinGrzegorz StarobratS FarrokhiGrzegorz TrybekS. Shahrukh HashmiGuan-Zheng LiuSabina Barrios-FernándezGuglielmo CampusSabine PirrGuido FillerSabine PlancoulaineGuido FitzeSafeera KhanGuido GembilloSaid ShamiehGuillermo PlazaSainath RamanGusztav BeltekiSally HoweGuy DecauxSalwa El El TawilGwo-Shing ChenSamantha J LainGyu-Hong ShimSamantha MaurottiH. Lester KirchnerSamantha MossHae-Sang LeeSamiksha WasnikHagen WulffSamira Sam-iee-ZafarghandyHala ChaabanSamira SoleimanpourHamadjam AbboubakarSamriddha RayHamdi ChtourouSamuel FurseHamid Yimam HassenSamuel Manzano-CarrascoHan KemperSamuel X. ShiHanifa BachouSanaa Najeh Al-Haj AliHank WhiteSandra SaavedraHanna LeeSandra TaylorHanna LiberskaSandro BrunelliHanna Przybyła-BasistaSandy K. WurteleHanna PułaczewskaSang Ho KwakHannah J. BrewerSanja Music MilanovicHannakaisa Niela‐VilénSanjay ChawlaHanne LademannSanjay KumarHanns MoshammerSantosh K. TadakamadlaHans Christoph LudwigSapna SharmaHans Ulrich BucherSara Anna BoniniHany AlySara CostanzoHarald EhrhardtSara E. WilliamsHarland S. WinterSara FilipiakHarold N. Lovvorn IIISara Frygner-HolmHarpreet KaurSara McLaffertyHarry IlandSarah B WallworkHasan AbedSarah E. RoseHasan JamalSarah K. MurnenHatem El-ShantiSarah LittleHatice AtilganSarah MasefieldHåvard LoråsSarah MoxonHayato GoSarah SpencerHeang LimSarah WarkentinHeather Gordish-DressmanSaral DesaiHeather J. BaldwinSarat ThikkurissyHeather RobertsSari AcraHeather SiefkesSasa MissoniHedwig DeubzerSathishkumar Ramalin-gamHeidi HillmanSaulius DrukteinisHeleen Muriel StaalSaumel AhmadiHelen BranthwaiteSayyed Ali SamadiHelen FisherScott CoussensHelen KoechlinScott M PickettHelen LeonardScott PeggHelen LileyScott SpreatHelen LittleSe Yong JungHelen PurtillSean BaileyHelen ShoemarkSebastian FarrHelen WoolleySebastian FrankeHelena MartynowiczSebastian G. WalterHendree E. JonesSebastian GehmertHendrik J. NiemarktSebastian GłowińskiHenri JustinoSebastian HoehlHenri TilgaSebastian WolfHenri Vähä-YpyäSebastiano LavaHenrik KöhlerSeda TierneyHenrique PereiraSeema KumarHenry C. LeeSeijun LimHenryk DudaSeil KimHerbert HallerSelma DemirHermien HartogSenthilkumar Sankarara-manHideaki KawabataSerena FossatiHideo WadaSerena SalomeHilary CaldwellSerge BrandHilary McClaffertySeverino Jefferson Ribeiro SilvaHilda MulrooneyShadi KhalilHillary Swann-ThomsenShahid BashirHimangshu SonowalShaista SafderHind AlsharhanShamim NematiHiran ThabrewShannon K. De L'EtoileHiroshi HoshijimaSharat Pani PaniHiroshi KurokiSheila RiggsHiroshi NakaseShel LevineHisayuki MiyagiSheng-Ling JanHo-Chang KuoShibani KanungoHolger MuehlanShigeki MatsubaraHolly A. RoyShinichi SuzukiHongwei YaoShinichiro MorichiHon-Yi ShiShinji TeramotoHoria CalniceanuShinji TsukamotoHortense PetatShinsuke MizutaniHrvoje BudinčevićShirin MadzhidovaHsueh-Yu LiShmuel ArnonHua-Fang LiaoShubhi KaushikHubertus HimmerichShun-Ku LinHubertus JochenShunsuke NosakaHue Jung Jung ParkShuping GeHueng-Chuen FanShyam K. SathanandamHugo SarmentoSigita BurokienėHui-Ling ChenSigne VahlkvistHülya ÖzsahinSihui MaHung-Lung ChiangSilvia CiminoHunter BennettSilvia LandiHuntington PotterSilvia Maya-EneroHwa Jin ChoSilvia PerzolliHyekyun RheeSilvia SpivakovskyIan BoggeroSilvio MaltagliatiIan HyslopSimon Morand-BeaulieuIan MartinsSimona CensiIanthi Maria TsimpliSimona MitevaI-Chan HuangSimone PisanoIgnasi Navarro SoriaSimone PratesiIgor DumicSinead RochaIgor P. KaidashevSisira EdirippuligeIker MadinabeitiaSi-Ton ChenIlan KatzSi-Tong ChenIlan SanfiSivakumar NuvvulaIlaria ChiricoSlawomir KozielIlaria RiboldiSławomir MurawiecIlias TsiflikasSnezhina LazovaIm Quah-SmithS??nia Maria Martins Car-idadeImeke GoldschmidtSofia O. MajorIna StephensSokou RozertaInas FarisSolomon OlumInês BaíaSonia Marzia GrandiInes TestoniSonia RobinsonInés VelascoSon-Nam NguyenInga SaitadzeSook-Hyun ParkInga VogesSophia CharitouInge SchmitzSophie DreamIn-geol HoSophie Rosa MerckaertInger KullSotirios FouzasIngvild Bruun MikalsenSoumeth AbasseInken BrockowSoumitri SilInmaculada C. Palo-mo-ToucedoSoyang KwonInmaculada Corral LiriaSoyoung KwakIoanna KosteriaSpyridon SiafisIoannis DelniotisSreekanth Kumar Mal-lineniIoannis KarakisSrinivas RamachandraIoannis SyrosStacy V. SmithIonela Ruxandra SfeatcuStavroula BarbounakiIonuț Isaia JeicanStavroula ParastatidouIraklis MourikisStefan Holland-CunzIrena FrycStefan J. FriedrichsdorfIrene CadimeStefan J. LangIrene Isabel P. LimStefan Kurath-KollerIris BergerStefan MihaicutaIsaac RheeStefan NilssonIsabel Cabrita CondessaStefania SetteIsabel Escobio-PrietoStefanie WeberIsabel MarziStefano GiulianiIsabella Felzer-KimStefano TozzaIsadora BeghettiStella StabouliIsmael MaatoukSten DreborgIsoken OlomuStephan PayrIsolde Martina BuschStephanie M. MoodyItaru YanagiharaStephanie M. MorrisIulia IoanStephanie M. WalshIvan Castilla-RodríguezStephen B. LevineIvan ChenchevStephen Kofi AninIvan HandStephen OnufrakIvan KopitovićStephen P. HawserIvan L. HandStephen R. DurhamIvan RadojaStephen Thaddeus Con-nellyIvan TrusStergios BoussiosIvana KholováSteve DuffinIvana TrivicSteven BischIzabela Małysz - CymborskaSteven D. BenderIzabela ZakrockaSteven G. KovenJ. Chase McNeilSteven KairysJ. D. G. KooijmanSteven M. DonnJ. Daniel TwelkerSteven M. SingerJaan ToelenSteven Ross MurrayJacek BoguckiStijn VerhulstJacek WilczyńskiStuart T HamiltonJackelyn Y. BoydenSubhransu SahooJackie BoydenSuhee KimJaclyn DyniaSune BoJacob I. FeldmanSung-kyu KimJacobus MullerSungmin KimJacqueline CurtisSunHee LeeJacqueline KentSusan FlynnJae Min LeeSusan J. GrosseJaesik ParkSusan MahanJaime DevineSusan NiermeyerJairo León-QuismondoSusan O'ConnellJakub SuchanekSusan TupperJames B. FinkSusana RodríguezJames CrowleySusann KobusJames D. GeyerSusanna Cunning-ham-RundlesJames LeeSusanne KobelJames O'Higgins NormanSusanne Martin HerzJames R. CouchSusanne R. KerscherJames R. MinerSusanne Ring-DimitriouJan Rafael DörrSusanne ThummlerJan Te NijenhuisSuzi TortoraJan Walter SchroederSuzy M. TeutschJana Van DyckeSven MichelJane ChudleighSvetlana TishkovskayaJane D. ChampionSwetha KanduriJane GaultneySy Duong-QuyJanelle SkinnerSyed Far Abid HossainJanez JazbecSylvia E. BadonJanieta BartzSylwia WiśniewskaJanine BernardoSyuji TakeiJanneke DekkerTadashi ItoJared R. RuchenskyTai-Heng ChenJareen Meinzen-DerrTaizo NakanoJarosław HerbertTakako MiyamuraJaroslaw PaluszczakTakao FujisawaJason BrumittTakaya HigakiJason C. YamTakayuki KishiJason GillilandTakehiko DoiJavier Adolfo De Las Heras MonteroTakemichi FukasawaJavier Almela-BaezaTakeshi KamiyaJavier De La CruzTakumi MitsuhashiJavier Flores-FraileTak-Wah WongJavier HerruzoTal-Saban MiriJavier Merino-AndresTamara SeidlJavier Ortuño SierraTamara YawnoJavier Rodríguez-FanjulTamás BerkiJavier Sánchez-SánchezTânia LucciJaya RachwaniTania ManoJayasree NairTanveer AhmadJean A. RondalTanya St. St. JohnJean Pierre KabueTara K. KaufmanJeanette GarciaTaru ManyangaJean-François BureauTatiana GurkoJean-Marie GraïcTatjana EwaisJeannette E. Dankert-RoelseTatjana RadosavljevićJeff CraneTayla PennyJeff GibbonsTaylor J. ReifJeffrey EckertTeija KoskelaJeffrey GagneTena NiseteoJeffrey GelferTeresa KruseJeffrey H. Cornelius-WhiteTerri P. McVeighJeffrey M. WilsonTerry KatzJelena JakabTerry R. LightJelena VekicTessel RigterJen-Fu HsuTetyana ChumakJenna R. CummingsThais Manzano ParisottoJennifer DiltsThayne L. SweetenJennifer Miner WeaverTheodora PapamitsouJennifer StinsonTheodore P. NicolaidesJennifer WalterThibaut ChapronJenny WilkinsonThomas AkintayoJens André HammerlThomas DavisJeong-won HanThomas E. EngelhardtJerzy ChudekThomas Franz KrebsJesse J. EschThomas J. AbramoJessica A. CasasThomas M AttardJessica A. ZagoryThomas M. AustinJessica BaconThomas R. WoodJessica GleasonThomas RaffayJessica HellingsThomas RiedelJessica RanieriThomas SabaJessica SnowdenThomas StewartJessika C. BolesThomas WenzelJesús DevesaTianji ChenJesus M. Martin-CamposTilman HumplJi Hoon SeoTilman HuppertzJiaan-Der WangTim JancelewiczJianhua MaoTina M SlusherJianxiong WangTina U CohnertJill M SwirskyTing FuJillian Vinall MillerTing-yi LinJim ButteryTiong Yang TanJing-Quan WangTobias Daniel TrippelJinyu XuTobias GorisJi-Yong JungTom De LeeuwJiyoung KimTom DuddingJo Andre Y. DensTom KovesiJoachim M. OertelTomasz DzierżanowskiJoan M. RomanoTomasz HanćJoana AchaTomasz UrbanowiczJoana AntunesTomaž VelnarJoanna BaranTommaso LombardiJoanna GarstangTomohiro HiradeJoanna KwiatkowskaTomoyuki KawamuraJoanna LawrenceToni HaapaJoanna TrelinskaTonia De GiuseppeJoao GuerreiroTonia VassilakouJoao JorgeTore Kristian AuneJoao RequichaTorleif RuudJoão SerranoToru WatanabeJoaquim CarrerasToshihiro TajimaJoaquim M. B. PinheiroToshimune KambaraJoaquim PinheiroToshinobu TakedaJocelyn E. RemmertTrevor GersonJochanan VeerbeekTrinidad GarcíaJoel E FraderTsang-Hai HuangJoelle KarlikTsuyoshi MatsumuraJoerg KlepperTsvetelina VelikovaJoern-Hendrik WeitkampTuan NguyenJohan LammensTudor Lucian PopJohan Vande WalleTuija TammelinJohan Von HeidekenTurcu-Stiolica AdinaJohanna BlomTyler J. KybartasJohanna FitzgeraldTzu-Yu PengJohannes MayrUdo RolleJohannes Van den AnkerUlrich WalterJohn B. CooperUndine LangJohn B. MorrisUrszula Krupa-KozakJohn EastwoodUsha KrishnanJohn GriniatsosUsha RajJohn Mark FroilandVaithinathan SelvarajuJohn MelvilleValentin ReutovJohn MondanaroValerio BonavolontaJohn RappVanesa Cantón-HabasJohn S. KimVanessa CavalleraJolanta ArtymVanessa Paredes GallardoJolanta Lis-KuberkaVanilson LemesJolanta ZuzdaVasanth KumarJolijn Van CauwenbergheVasileios BekiarisJonatan Barrera-ChimalVasiliki ChondrogianniJonathan K. FernandVasu D. GootyJonathan MosesVaughns JanelleJong-woon ChoiVendela ZetterqvistJoonyoung LeeVenturella RobertaJordi MogasVeronica Alonso-ArroyoJörg RichterVeronica DusselJorge Amate-RomeraVeronica LuqueJor̈n Hendrik WeitkampVerónica Martínez-LópezJörn-Hendrik WeitkampVéronique KemmelJose Boix-OchoaVicky LehmannJosé D. UrchagaVictoria M. AtzlJosé Javier Zamorano-LeonVikash AgrawalJose L. PeiroVikramaditya DumpaJosé Luis Romero-BejarVinay SharmaJosé MendesVinayak K. NaharJosé Pablo Miramon-te-GonzálezVincent D. GaertnerJosé SilvaVincenzo SalvoJose Tomas Ramos Ama-dorVincenzo ZanonJosefa González SantosVineet BhandariJoseph GiganteViola OldratiJoshua D. RobinsonVirginie NicaiseJoško MarkićVito PavoneJost KaufmannVittoria Carnevale PellinoJovan GardasevicVittorio ChecchiJovan LovrenskiVivek A. BhadriJozsef KatonaVlad Laurentiu DavidJuan Antonio Valera-CaleroVladimir W. SpolskyJuan Bautista De SanctisVo Truong Nhu NgocJuan Carlos López-GarcíaVolker SchöfflJuan Carlos SierraVon Hofsten ClaesJuan José DíazWai Yew YangJuan M. García-CeberinoWaiz WaseyJuan Vega-EscañoWaldemar A. CarloJudith MercerWalter K. KraftJu-Lee OeiWalter R. SchummJulia Guerrero-GironésWannasiri LapcharoensapJulia Haba-OscaWei-Ting HsuJulia MoosmannWeiyan YinJulian Nevado BlancoWelch KathrynJuliana PuppiWen LinJuliane VölkerWenceslao PeñateJuliet Haarbauer-KrupaWendla SensingJulio Alvarez PittiWendy SaltzmanJumpei SaitoWenhao ZhouJun KameiWen-Ko ChiouJun KohyamaWenzhen GeJun SuzukiWidjane Sheila Ferreira GoncalvesJun WatanabeWilbert P. VermeijJungHun ChoiWilhelm Robert GroszJunichiro TezukaWillem Van De VeenJun-O JinWilliam MorelloJurrianne C. FahnerWilliam RaffaeliJustyna ModrzejewskaWilliam T. ZempskyJustyna Opydo-SzymaczekWilrike J. PasmanJuyeong KimWolf RogowskiKaat DesloovereWouter CoolsKai ErenliWynne E. MorrisonKai KarosX. DelforgeKai KönigXiangli GuKaitlin A. OswaldXiangli ZhaoKalika PaigeXiangpeng LiaoKalliopi MegariXiao-Li WangKamel GanaXiaoyue PanKameron ModingXin TongKamila Czepczor-BernatXiuyun WuKamila Pokorska-NiewiadaYael LebenthalKandice KapinosYafit HirshlerKang-soo LeeYanhong HuangKarel AllegaertYanjun RenKaren BaxYannis DotsikasKaren ChoongYao-Chuen LiKaren Dineen WagnerYasmin BainsKaren MeltonYasser SolimanKaren RotabiYen-Ju ChuKaren SpruytYen-Lin LiuKaren ThorpeYian ZhangKaren WebbYi-Chen HsiehKari NielsenYing-Chi LinKarl AndriessenYing-Hsien HuangKarla Marie FredricksYin-Hsiu ChienKarleen GribbleYitzchak FrankKarthik RagunathanYi-Wen ChiuKaryn J RobertsYohei IkezumiKasper SalinYonas BekeleKatarzyna BergmannYong Hwan KimKatarzyna DylewskaYongsuk SeoKatarzyna KaczyńskaYoonJung LeeKatarzyna KorybalskaYoshiharu KawaguchiKatarzyna ŁubiechYoshimitsu FujiiKatarzyna Moc-ny-PachońskaYoshio NakataKatarzyna RygielYoshitomo YasuiKatarzyna Zab-orowska-SapetaYou-Lin TainKate FrazerYoung-jae KimKatelynn E. BoernerYoung-Joo SimKath PetersYu Wei ChenKatharina DiehlYueqi YanKatharina Korecky-KröllYuexin XuKatharina RufenerYuh-Jyh LinKatharina SchuhmannYuichi TakashiKatharine SarikakisYuji ItoKatherine DarlingYu-Ling ChenKatherine KochYun HanKatherine S. SalamonYung-Chung ChenKathleen LemanekYung-Sheng ChenKathryn Ann Kelly McQueenYusuke OkuboKathryn E. KylerYvonne ZurynskiKathryn G. AndrewsZachary VesoulisKathryn K. OstermaierZachary Wahl-AlexanderKathryn Woods-TownsendZbigniew KulagaKathy J. JenkinsZbigniew ZyliczKatie F. LovesonŽeljko AntićKatja JoronenZenon PogorelićKatja KousZhenqiu LiuKatja ParschatZhi WangKatrin D. Bartl-PokornyZhi-Fu WuKatrina HutchinsonZiba VaghriKaustuv BhattacharyaZoe KnowlesKavita Desai DakojiZoltan AntalKazuhiko KotaniZygmunt WarzechaKazumichi Fujioka



